# Phylogenetic analysis reveals diversity in glycan biosynthesis in “*Candidatus* Accumulibacter”

**DOI:** 10.1016/j.bioflm.2026.100350

**Published:** 2026-02-05

**Authors:** Simon A. Eerden, Thomas Abeel, Mark C.M. van Loosdrecht, Samarpita Roy

**Affiliations:** a Department of Biotechnology, Delft University of Technology, Van der Maasweg 9, Delft, 2629 HZ, Netherlands; b Department of Intelligent Systems, Delft University of Technology, Van Mourik Broekmanweg 6, Delft, 2628 XE, Netherlands; c Section of Environmental Microbiology, Aalborg University, Fredrik Bajers Vej 7H, Aalborg, 9220, Denmark

## Abstract

Although biofilms are widespread in nature, the ecological roles and compositional diversity of the extracellular polymeric substances (EPS) forming these structures remain poorly understood. Here, we apply a bottom-up genomic approach by investigating the biosynthetic potential for glycan precursors in the genus “*Candidatus* Accumulibacter”, with a focus on assessing the intra-genus variability. Within a curated set of 61 “*Ca.* Accumulibacter” MAGs, our analysis revealed a dichotomy in glycan precursors between a conserved core group of 9 nucleotide-sugars and a variable accessory set of 12 nucleotide-sugars, out of 50 nucleotide-sugars tested. The core nucleotide-sugars in “*Ca*. Accumulibacter” are related to nucleotide-sugars also found to be widely distributed across the tree of life, whereas the accessory set is enriched in rare nucleotide-sugars. The accessory nucleotide-sugars show an irregular distribution across “*Ca.* Accumulibacter” phylogeny, and divergent evolutionary histories. This highlights the possibility that distinct evolutionary pressures act on different parts of the EPS-formation metabolism, leading to genotypic diversification driven by complex biological phenomena such as horizontal gene transfer that support the observed divergent evolutionary histories.

## 1 Introduction

It has been estimated that 40-80% of all bacteria live in biofilms [1]. Some even propose the idea that biofilms are the default mode of microbial life, with the widely studied planktonic cultures being *in vitro* artifacts resulting from cultivation bias [2]. The high prevalence of biofilms in natural environments underscores the importance of the biofilm mode of growth for microbial ecology. Biotechnological processes, such as wastewater treatment plants, often rely on microorganisms embedded within biofilms. However, in such systems, the ecological and physiological principles underlying the production of the biofilm matrix are rarely studied in depth.

Microbes construct the biofilm matrix by excreting *Extracellular Polymeric Substances* (EPS), embedding themselves in a hydrogel-like material majorly composed of proteins and glycans [3]. At the molecular level, these polymers display a high degree of chemical heterogeneity [4]. In addition, the composition of EPS depends on the species living inside the biofilm, as well as on the environmental conditions. In one hand, this variability arises from differences in the presence and usage of microbial EPS biosynthesis pathways. On the other hand, chemical modifications of the EPS matrix could also occur in the extracellular space after its production, allowing for dynamic finetuning of an existing EPS matrix [[5], [6], [7]].

Omics-based approaches can be useful to shed light on the EPS constituents and the genomic potential for their biosynthesis. Using metaproteomics-based approaches, putative structural EPS proteins can be identified in biofilm samples [8]. However, studying glycans is significantly more complicated since their structures are not derived from a directly readable template in the genome. Instead, their structures arise from the specificity of dedicated enzymes, most importantly glycosyltransferases, that are notoriously hard to infer from sequence alone. Previously, a metagenomics-based approach has been employed to search for polysaccharide gene clusters in wastewater metagenomes [9]. However, this approach specifically queries gene clusters related to previously identified glycans, whereas a vast portion of the glycome and its corresponding genes remain unidentified. Therefore, this top-down approach may not have a broad coverage of the glycome and provide an incomplete view of its diversity.

To probe the genetic potential for glycome biosynthesis, a bottom-up metagenomics-based approach might be useful as well. We hypothesise that glycosyltransferases depend on nucleotide-sugar building blocks; therefore, evaluating the biosynthetic potential for monomers in microorganisms provides key insight into EPS composition. Unlike amino acids and nucleotides, the alphabet of available nucleotide-sugars is not universal in biology [10]. Genes for nucleotide-sugar biosynthesis pathways are therefore often encountered in gene clusters for glycan biosynthesis [11]. Consequently, variations in nucleotide sugar biosynthesis likely also reflect variability in the wider glycome.

The glycome of biofilms in microbial ecosystems has thus far been poorly described, with most well-studied biofilm systems limited to pure cultures, typically in clinical settings. In microbial ecosystems, EPS have been shown to help microorganisms adapt to changes in redox conditions, availability of nutrients, predation, or desiccation. One such ecosystem where EPS production plays a significant role is in wastewater treatment plants. In these processes, EPS promotes clean water production by facilitating biomass separation through flocculation and biofilm or granule formation. Meanwhile, EPS harvested from such processes are increasingly studied as a source of renewable biopolymers for industrial applications, such as flame retardants [12], bioplastics [13] and seed coatings [14]. The genus “*Candidatus* Accumulibacter” is a key functional taxon in wastewater treatment plants and has been extensively studied in terms of its physiology [15,16] and is increasingly studied for EPS production [17,18]. Yet, a comprehensive picture of its glycan profile remains lacking. Therefore, our aim was to explore the genomic potential for the production of the glycan component of EPS in the genus “*Ca.* Accumulibacter”, assess its intra-genus variability, and evaluate the relationship between potential glycan type and phylogenetic diversity.

## 2 Methods

### 2.1 Curation of MAG database

A dataset of 118 MAGs classified as “*Ca*. Accumulibacter” was retrieved from the NCBI database. The quality of the MAGs was scored using CheckM v1.2.2 [19], and all MAGs with <90% completion and >5% contamination were removed from further analysis. A taxonomic tree of the MAGs adopting the latest redefinition of members belonging to the genus “*Ca*. Accumulibacter” was generated based on the *ppk1* gene, as proposed by Petriglieri et al. [20]. To that end, we searched for the *ppk1* gene with HMMER, using the KEGG KOFAM profile for *ppk1* (KO number: K00937). MAGs that did not return hits were discarded. In case that multiple hits were found, only the hit with the highest bitscore was used. The detected *ppk1* sequences were then aligned using MAFFT v7.505 [21]. A tree was constructed from this alignment using FastTree version 2.1.11 [22], using default settings. The resulting phylogenetic tree was rooted with the *ppk1* sequence from *Dechloromonas aromatica* (accession number: CP000089.1) as outgroup. All MAGs not sharing the last common ancestor of ”*Ca.* Accumulibacter regalis” (a clade I species) and ”*Ca.* Accumulibacter similis” (a clade II species) were discarded, as they fall outside the existing taxonomic framework for “*Ca.* Accumulibacter” and might therefore not give useful phylogenetic insights. An overview of this curation process, as well as the accession numbers of the used MAGs, can be found in Supplementary Table S1.

### 2.2 Gene and pathway detection

To search for gene sequences in the MAGs, HMMER 3.3.2 was used [23]. From each MAG, ORFs were first identified and translated using prodigal v2.6.3 [24]. The resulting sequences were then passed to HMMER using the hmmsearch command using default settings. The used HMM profiles were retrieved from the KEGG KOfam database, version 2023-11-01 [25], from which we selected all HMM profiles corresponding with KO numbers connected to the “Biosynthesis of Nucleotide Sugars” map (map01250). To minimise false positives resulting from poorly supported models, HMM profiles corresponding to KO entries with fewer than 200 known sequences in the KEGG database were excluded from analysis. As reporting thresholds, the values were chosen as recommended by KEGG. In the case that no threshold was specified, an e-value cutoff of 1e-20 was used, combined with a query cover threshold of at least 75%. All genes that were detected in 90% of the MAGs or more were classified as core genes, while the other detected genes were denoted as accessory genes.

To determine whether a pathway to a particular nucleotide-sugar was present as a whole, we used two criteria for detection that were both required to be true: (i) there must be a 75% complete network connection to a central carbon metabolism intermediate (fructose-6-phosphate, ribulose-5-phosphate or sedoheptulose-7-phosphate) and (ii) the terminal enzyme in the pathway must be present. Nucleotide-sugars for which a pathway has been detected in more than 90% of the MAGs were classified as core nucleotide-sugars, while the other detected monomers were classified as accessory nucleotide-sugars.

### 2.3 Monophyly analysis of gene families

To analyse the monophyletic or polyphyletic tendencies of each gene family in ”*Ca.* Accumulibacter”, we followed an adaptation from the procedure by Zhaxybayeva et al. [26]. For each gene family, 200 randomly sampled examples were downloaded from the KEGG database. Using FastTree (version 2.1.11), phylogenetic trees of each family were then constructed from these sequences, combined with the sequences detected in the ”*Ca.* Accumulibacter” MAGs. The degree of monophyly was scored by the fraction of ”*Ca.* Accumulibacter” sequences that covers the largest monophyletic clade, where a value of 1 represents perfect monophyly, and lower numbers represent a more scattered distribution. Additionally, gene families in which the support for the last common ancestor of all ”*Ca.* Accumulibacter” genes was below 80% were excluded from analysis, as they do not contain reliable phylogenetic information.

### 2.4 Data analysis

For data analysis, python (v3.10.13) was used. Permutation tests were used to test the significance of differences (monophyly, gain/loss events and nucleotide-sugar prevalence in the bacterial kingdom) between the core and accessory groups, using the difference in medians between the two groups as test statistic. The permutation test was performed using the permutation_test function from the scipy package, using standard settings, except for setting the number of permutations at 100000.

The minimum number of gain or loss events required to obtain an observed presence/absence pattern of a pathway given the *ppk1* tree was calculated using Fitch's algorithm [27].

## 3 Results

### 3.1 The “*Ca.* Accumulibacter” genus has a variable nucleotide-sugar metabolism

**Fig. 1:**
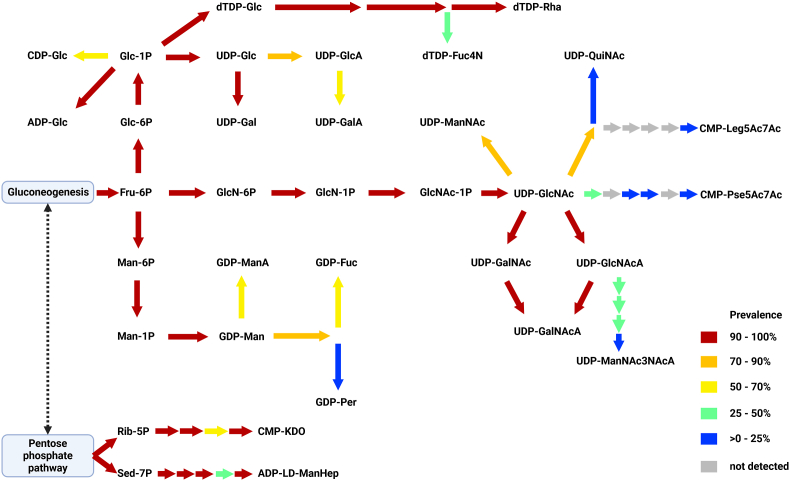
**“*Ca.* Accumulibacter” species have varying potential for glycan precursor biosynthesis.** Reactions in the biosynthetic metabolism of nucleotide sugars are indicated as separate arrows. The color of each arrow indicates the prevalence of that reaction in the set of 61 “*Ca.* Accumulibacter” MAGs analysed in this study. Prevalence values can be found in Supplementary Data S2. (Abbreviations, dTDP-Glc: dTDP-glucose, dTDP-rha: dTDP-rhamnose, dTDP-Fuc4N: dTDP-4-amino-4,6-dideoxy-galactose, CDP-glc: CDP-glucose, ADP-Glc: ADP-glucose, UDP-Glc: UDP-glucose, UDP-Gal: UDP-galactose, UDP-GlcA: UDP-glucuronic acid, UDP-GalA: UDP-galacturonic acid, UDP-GlcNAc: UDP-N-acetyl-glucosamine, UDP-GalNAc: UDP-N-acetyl-galactosamine, UDP-ManNAc: UDP-N-acetyl-mannosamine, UDP-GlcNAcA: UDP-N-acetyl-glucosamine uronic acid, UDP-GalNAcA: UDP-N-acetyl-galactosamine uronic acid, UDP-ManNAc3NAcA: UDP-2,3-diacetamido-2,3-dideoxy-d-mannuronic acid, UDP-QuiNAc: UDP-N-acetyl-quinovosamine, CMP-Pse5Ac7Ac: CMP-pseudaminic acid, CMP-Leg5Ac7Ac: CMP-legionamic acid, GDP-Man: GDP-mannose, GDP-ManA: GDP-mannuronic acid, GDP-Fuc: GDP-fucose, GDP-Per: GDP-perosamine, CMP-KDO: 3-deoxy-d-manno-octulosonate, ADP-LDManHep: ADP-l-*glycero*-beta-d-manno-heptose). (For interpretation of the references to color in this figure legend, the reader is referred to the Web version of this article.)

To explore the degree of variation in genomic potential for nucleotide-sugar biosynthesis in the “*Ca*. Accumulibacter” genus, we searched a curated set of MAGs for the responsible metabolic enzymes and mapped out their presence in the metabolic network (Fig. 1). This analysis reveals a core set of nucleotide-sugar-related genes in the “*Ca.* Accumulibacter” genus. These core nucleotide-sugar genes are responsible for the biosynthesis of simple hexoses, hexosamines, hexosamine uronic acids, and the deoxy sugar rhamnose. By contrast, an approximately equal number of nucleotide-sugar genes exhibits variable presence across smaller subsets of “*Ca.* Accumulibacter” species. The products of these accessory nucleotide-sugar pathways include a wider variety of sugars, such as uronic acids, nonulosonic acids, and complex sugars such as N-acetylquinovosamine, perosamine or 2,3-diacetamido-2,3-dideoxy-d-mannuronic acid.

Interestingly, the core nucleotide-sugar pathways form a contiguous network connected to the central carbon metabolism, while the accessory nucleotide-sugar pathways branch out from this core. The presence of such a structural organisation suggests that the observed variability is not a mere artifact of incompletions in MAGs. Instead, it may reflect genuine differences in metabolic potential among different “*Ca.* Accumulibacter” species.

**Fig. 2:**
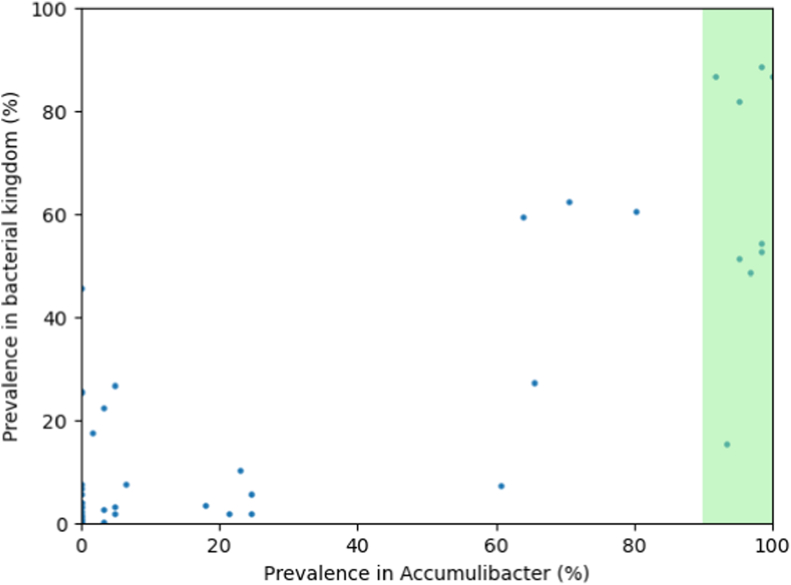
**Accessory nucleotide-sugars in the “*Ca.* Accumulibacter” genus tend to be less common in the bacterial kingdom.** Each point represents a nucleotide-sugar. The x-axis indicates the prevalence of that nucleotide-sugar in the set of 61 “*Ca.* Accumulibacter” MAGs analysed in this study. The prevalence of the same nucleotide-sugar throughout the bacterial kingdom is shown on the y-axis, according to Ref. [10]. The green shading highlights the core nucleotide-sugars in “*Ca.* Accumulibacter”. (For interpretation of the references to colour in this figure legend, the reader is referred to the Web version of this article.)

The prevalence of the detected pathways in “*Ca.* Accumulibacter” was then compared to their general prevalence across the bacterial kingdom (Fig. 2, Supplementary Table S2), using the dataset of Srivastava et al. [10]. A permutation test reveals that the set of core nucleotide-sugar pathways is significantly enriched in commonly occurring monosaccharides compared to the accessory set (*p* = 0.0029). This comparison establishes a strong correlation between the variability of nucleotide-sugar presence within the “*Ca.* Accumulibacter” genus and the bacterial kingdom in general.

### 3.2 Nucleotide-sugar genomic potential is irregularly distributed among “Ca. Accumulibacter” phylogeny

**Fig. 3:**
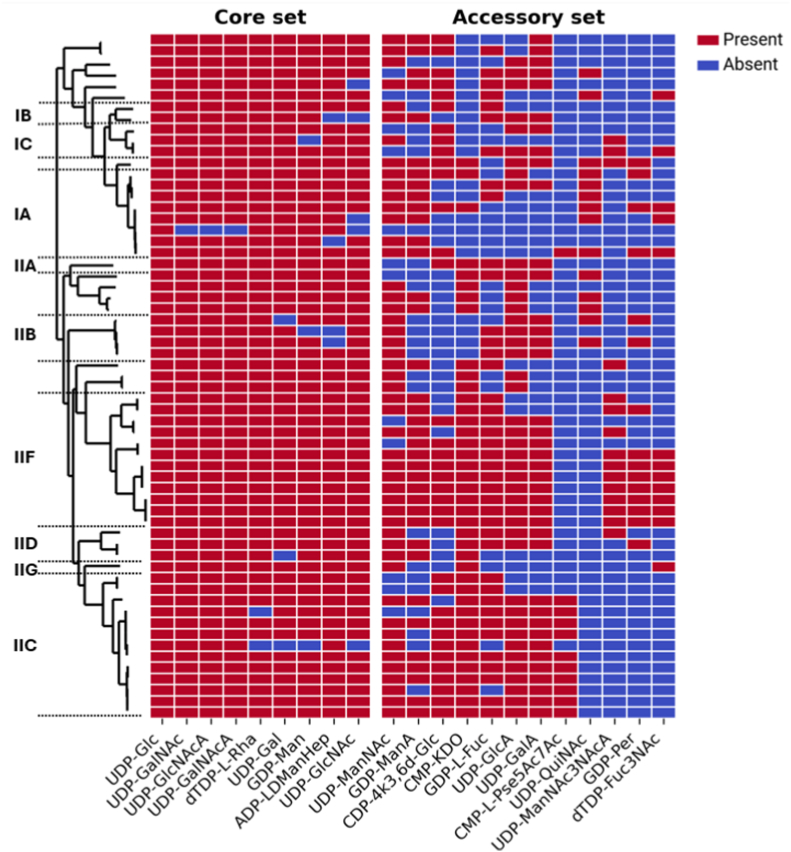
**The presence of accessory nucleotide-sugar biosynthesis pathways does not show phylogenetic coherence across the “***Ca.***Accumulibacter” genus.** The *ppk1*-based taxonomy of the 61 “*Ca.* Accumulibacter” MAGs used in this study is shown by the dendrogram on the left side. Each row in the heatmap corresponds to one of the leaves of that tree. The presence of a nucleotide-sugar biosynthetic pathway is indicated by a red colour in the corresponding column. Absence of a pathway is marked with a blue colour. (For interpretation of the references to colour in this figure legend, the reader is referred to the Web version of this article.)

“*Ca.* Accumulibacter” is a phylogenetically diverse group, with an ANI score of 85% [20]. The observed degree of variability in potential glycan precursor pathways raises the question whether these variations are taxonomy-related. To that end, we compared the presence of nucleotide-sugar pathways to the taxonomy of “*Ca.* Accumulibacter” based on the phylogeny of the *ppk1* gene as proposed by Petriglieri et al. [20] (Fig. 3). Our analysis indicates that a set of core nucleotide-sugars is present across all “*Ca.* Accumulibacter” MAGs whereas the accessory nucleotide-sugars are not conserved phylogenetically. For many of the accessory nucleotide-sugars, we observe that their presence tends to follow a polyphyletic pattern with respect to the *ppk1* phylogeny. To quantify the irregularity of the presence patterns, we used Fitch's algorithm, calculating the minimum number of independent gain or loss events required to get the observed pattern. According to this method, a median of 10.5 gain or loss events (interquartile range 7 – 11.75) was required to explain the observed patterns in the accessory nucleotide-sugar pathway set, compared to 2 gain or loss events (interquartile range 1 – 3.5) for the core nucleotide-sugar pathways (Supplementary Table S3). Using a permutation test, the accessory group was found to have a significantly higher number of gain and loss events than the core group (p = 0.0008), highlighting that the accessory nucleotide-sugars exhibit a more polyphyletic distribution, likely shaped by frequent and independent evolutionary events.

### 3.3 Accessory nucleotide-sugar pathways have divergent evolutionary histories

**Fig. 4:**
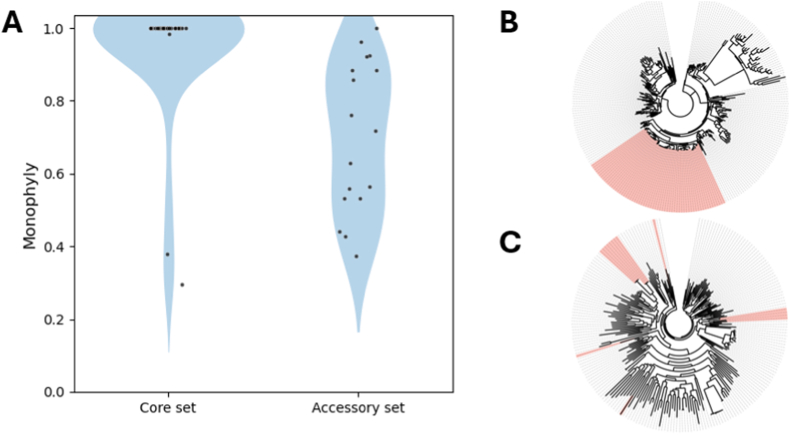
**Accessory nucleotide-sugar gene families in “*Ca.* Accumulibacter” show different evolutionary histories than core nucleotide-sugar gene families. A)** “*Ca.* Accumulibacter” core nucleotide-sugar genes are notably more monophyletic in their gene families than accessory nucleotide-sugar genes. Individual gene families are represented as dots within a density function (blue shade). The degree of monophyly is shown on the y-axis. **B and C)** Example phylogenetic trees of a typical core nucleotide-sugar gene family (B: UTP-glucose-1-phosphate uridylyltransferase, K00963) and a typical accessory nucleotide-sugar gene family (C: UDP-N-acetyl-alphaD-quinovosamine dehydrogenase, K24310). The leaves shaded in red indicate sequences found in the analysed “*Ca.* Accumulibacter” MAGs. (For interpretation of the references to colour in this figure legend, the reader is referred to the Web version of this article.)

The observed inconsistency between the presence of the accessory nucleotide-sugars and the ppk1-based taxonomy raises the question to what degree the responsible genes are related to each other. The relatively high number of inferred gain and loss events led to the hypothesis that the evolutionary histories of homologous accessory genes might not overlap within our “*Ca.* Accumulibacter” dataset. To test this hypothesis, we analyzed to what extent the detected sequences from the “*Ca.* Accumulibacter” MAGs form a well-supported monophyletic group within their respective gene families (Fig. 4).

Interestingly, core nucleotide-sugar genes typically show absolute monophyly within their gene families, with a median monophyly score of 1 (interquartile range 1 - 1), meaning that they all share a common ancestor in “*Ca.* Accumulibacter”. The only observed exceptions are all related to the biosynthesis pathway of dTDP-rhamnose, which is missing in only 2 of the 61 MAGs analyzed in this study. In stark contrast, the accessory nucleotide-sugar genes tend to form polyphyletic groups, with a median monophyly score of 0.72 (interquartile range 0.53 - 0.88), meaning that there are several independent evolutionary lineages of these genes in the “*Ca.* Accumulibacter” genus. Using a permutation test, we found that the separation in monophyly score between core and accessory genes is highly significant (p = 0.0002). This highlights that the typical evolutionary history of a core nucleotide-sugar pathway in “Ca. Accumulibacter” is fundamentally different from that of an accessory nucleotide-sugar pathway.

## 4 Discussion

### 4.1 The genetic potential for glycans exhibits high variability

In this study, we identified the genomic potential of “*Ca.* Accumulibacter” to produce 21 different nucleotide-sugars, 12 of which are accessory nucleotide-sugars. It is interesting to note that many of these glycosyl components, such as glucose, galactose, mannose, N-acetyl-glucosamine, rhamnose, fucose, glucuronic acid, galacturonic acid and N-acetyl-quinovosamine have previously been identified in the EPS of “*Ca.* Accumulibacter” enrichment cultures [28,18], providing experimental support for the targeted analysis of glycome precursor pathways. A similar analysis had previously been employed by Srivastava et al. [10], aimed at the degree of variability of glycan precursor biosynthesis across the entire prokaryotic domain. The authors found high variability throughout the prokaryotic domain. They also noticed that considerable variability could exist between different strains of the same species, which is consistent with our own observations. Yet, the evolutionary aspect of how these biosynthetic genes were related between species was not within the scope of their work, while it is a focus in this study.

The dependency of glycan biosynthesis on nucleotide-sugar production, as well as the tendency of nucleotide-sugar genes to form gene clusters with glycan biosynthesis machineries [11,29], strongly suggests that the genomic potential for whole glycans includes at least a similar degree of variability between closely related species. Still, many gene clusters exist that would not be covered by our search method, as they do not contain nucleotide-sugar biosynthesis genes. For example, the *gum* operon for xanthan production in *Xanthomonas campestris* does not contain nucleotide-sugar biosynthesis genes, but instead depends on other operons for precursor supply [30]. Therefore, it remains to be observed directly to what extent the complete biosynthetic machinery, including glycosyltransferases and other glycan-related enzymes, exhibits variability between closely related species. Some attempts have been made to search for glycan biosynthetic gene clusters in their entirety rather than only targeting precursor genes. For example, Dueholm et al. [9] employed a search algorithm relying on the presence of a combination of genes in sequential proximity and applied it on a database of MAGs retrieved from various Danish active sludge WWTPs. Using this method, the authors identified the production of PNAG, which is a polymer of only (N-acetyl-) glucosamine, in 2 “*Ca.* Accumulibacter” MAGs. Still, some limitations exist that should be overcome before a strategy like this can be applied to study the full variability of the biosynthetic potential of the glycome. These challenges include the combinatorial explosion of the number of possible glycans from the same building blocks, and the corresponding number of associated gene clusters, requiring a vast number of experimentally validated example gene clusters to gain broad coverage, while this information is currently scarce. Additionally, our limited ability of inferring glycosyl transferase activity, coupled to the high sensitivity of glycosyltransferase specificity to small mutations [31], complicates the task of functionally distinguishing these gene clusters.

### 4.2 Core and accessory nucleotide-sugars may serve different roles

In this study, a striking dichotomy was revealed between a core set of nucleotide-sugars, as opposed to an accessory set of nucleotide-sugars. The core set of nucleotide-sugars consists of common monosaccharides, of which the genes generally follow monophyletic trends in their gene families. By contrast, the accessory set of nucleotide-sugars consists of rare monosaccharides that exhibit large numbers of presence gain/loss events, while the detected genes for these nucleotide-sugar are also polyphyletically distributed within their gene families. This suggests a more complex evolutionary history for the accessory nucleotide-sugar set than for the core set.

The observed differences between the core and accessory nucleotide-sugar sets may reflect distinct evolutionary pressures, suggesting that these groups of monosaccharides serve different functions. The close conservation of the core nucleotide-sugar pathways further suggests that they are essential to sustain normal physiology in “*Ca.* Accumulibacter”. This is also supported by the observation that the core nucleotide-sugar pathways form a contiguous skeleton of pathways connecting to the central carbon metabolism, since missing enzymes in this group would likely disable multiple downstream pathways. By contrast, the polyphyly of the accessory nucleotide-sugars, and their peripheral location in the nucleotide-sugar metabolic network, suggest a more situational functionality.

### 4.3 Co-evolution could drive glycome diversification

The observed variability raises the question of what selective pressures could be shaping the glycome. Different monomer compositions can alter physicochemical and mechanical properties of the resulting polymers, for example through the charge density [32]. As glycans form the interface between the cell and the environment, shifts in the abiotic environment may change the need for glycans with different physicochemical properties. However, many “*Ca.* Accumulibacter” MAGs have been obtained from enrichment cultures. These are typically operated under similar conditions, for example with an anaerobic/aerobic cycle, anaerobic feeding of volatile fatty acids and high doses of phosphate [[33], [34], [35]], and therefore, a large diversity would not be expected. Consequently, abiotic factors are unlikely to be the sole driver for the observed variability between the tested MAGs.

This raises the possibility that biotic interactions play an important role in diversifying the glycome. In such interactions, the selective advantage of a genotype can depend on the abundance of other genotypes, a phenomenon known as frequency-dependent selection (FDS). Of particular interest in this context is negative FDS, where a genotype becomes less favourable as it increases in abundance. Such dynamics are known to drive genotypic diversification [36]. Since the glycome, especially the lipopolysaccharide (LPS) fraction, is involved in molecular recognition between species, the glycome could therefore also be subject to FDS. For example, bacteriophages use bacterial LPS to recognise and infect bacteria, driving bacteria to diversify their LPS structure to lower the vulnerability of the whole population to a phage epidemic [37]. Similar dynamics exist in pathogen-immune system interactions [38], as well as between bacteria and protozoan predators [39]. Additionally, negative FDS can arise from mutualism as well. For example, the symbiotic recognition between plants and *Rhizobium* species exhibits negative FDS to exclude inefficient nitrogen fixers from nodulation [40,41]. Thus, a mechanism involving negative FDS related to molecular recognition could be a plausible explanation for the observed variability in the “*Ca.* Accumulibacter” glycome.

### 4.4 HGT may play a role in shaping the glycome

Aside from the selective pressure, another key question is how the observed variability evolves from a genetic point of view. Gene duplication and loss within “*Ca.* Accumulibacter” alone are unlikely to explain the observed diversity, because the accessory nucleotide-sugar genes often have different evolutionary histories. Instead, horizontal gene transfer (HGT) might better explain the observed diverse evolutionary history.

Previously, HGT has been reported specifically for LPS O-antigen gene clusters in multiple organisms, such as *Bordertella*, *E. coli* and xanthomonads [[42], [43], [44]]. Moreover, EPS-related operons have been found on plasmids [45], indicating that an evolutionary pressure can exist for biofilm-related genes to become mobile. These observations raise the open questions of what part of the wider glycome of an organism is deterministically predictable by species taxonomy, and what part depends on a stochastic environmental gene pool. To answer this question will require future research directed towards identifying and analysing plasmids and other mobile elements specifically for EPS-related operons and LPS O-antigen gene clusters.

### 4.5 Outlook

Our findings not only improve fundamental understanding of glycan ecology, but it may also have biotechnological implications. Our phylogenomic analysis identifies the presence of conserved core nucleotide sugar pathways across the across *Ca. Accumulibacter* genus, suggesting that a fraction of EPS composition may inherently be robust and predictable. In contrast, the accessory pathways associated with rare monosaccharides can introduce inherent unpredictability in EPS composition. If biotic interactions, co-evolution, and mobile genetic elements drive variability in accessory pathways, then the glycan composition may be difficult to control by growth conditions or process design, due to stochastic selective pressures.

Consequently, for EPS recovery from open cultures such as wastewater treatment processes, this implies that product consistency may depend primarily on the contribution of the conserved glycan fraction, while the variable component may fluctuate due to biotic interactions or evolutionary dynamics. On the other hand, it should also be noted that a large variability in genomic potential does not automatically translate into proportional variability in phenotype. The mass fraction and functional contribution of the variable component of produced EPS remains unknown. It remains an open question for industrial applications in EPS-based resource recovery whether quantifying the contribution of the variable EPS component should be the focus, or EPS should be characterised from a broader physicochemical standpoint.

## 5 Conclusion

Overall, this study shows that approaching the glycome from the perspective of the biosynthesis of its building blocks can provide new insights in the wider variability of the EPS. The presence of an accessory set of pathways with diverging evolutionary histories within the genus of “*Ca.* Accumulibacter”, as opposed to an evolutionarily conserved core set, highlights the importance of the evolutionary dynamics acting on the glycome. Future studies should focus on further mapping the extent and impact of this variability in greater detail, as well as on identifying the selective pressures acting on the genomic potential for EPS biosynthesis within microbial ecosystems.

## CRediT authorship contribution statement

**Simon A. Eerden:** Writing – original draft, Visualization, Validation, Methodology, Investigation, Formal analysis, Data curation, Conceptualization. **Thomas Abeel:** Writing – review & editing, Conceptualization. **Mark C.M. van Loosdrecht:** Writing – review & editing, Supervision, Funding acquisition, Conceptualization. **Samarpita Roy:** Writing – review & editing, Methodology, Funding acquisition, Conceptualization.

## Funding

This research was supported by the 10.13039/501100009708Novo Nordisk Foundation (REThiNk, Grant 10.13039/501100009708NNF22OC0071498). **Samarpita Roy** was supported by the 10.13039/501100000780European Union's 10.13039/100018693Horizon Europe research and innovation program under the Marie Skłodowska-Curie grant agreement No 101068900. **Mark van Loosdrecht & Samarpita Roy** were supported by the SIAM Gravitation Grant 024.002.002, The Netherlands Organization for Scientific Research.

## Declaration of competing interest

The authors declare that they have no known competing financial interests or personal relationships that could have appeared to influence the work reported in this paper.

## Data Availability

The used python scripts have been uploaded to the following github repository: https://github.com/SiemEerden/Accumulibacter_glycan_variability_analysis.

## References

[1] Flemming H.C., Wuertz S. (2019). Bacteria and archaea on Earth and their abundance in biofilms. Nat Rev Microbiol.

[2] Jefferson K.K. (2004). What drives bacteria to produce a biofilm?. FEMS (Fed Eur Microbiol Soc) Microbiol Lett.

[3] Seviour T., Pijuan M., Nicholson T., Keller J., Yuan Z. (2009). Understanding the properties of aerobic sludge granules as hydrogels. Biotechnol Bioeng.

[4] Seviour T., Derlon N., Dueholm M.S., Flemming H.C., Girbal-Neuhauser E., Horn H., Kjelleberg S., van Loosdrecht M.C.M., Lotti T., Malpei M.F., Nerenberg R., Neu T.R., Paul E., Yu H., Lin Y. (2019).

[5] Little D.J., Bamford N.C., Pokrovskaya V., Robinson H., Nitz M., Howell P.L. (2014). Structural basis for the De-N-acetylation of poly-β-1,6-N-acetyl-D-glucosamine in gram-positive bacteria. J Biol Chem.

[6] Rozeboom H.J., Bjerkan T.M., Kalk K.H., Ertesvåg H., Holtan S., Aachmann F.L., Valla S., Dijkstra B.W. (2008). Structural and mutational characterization of the catalytic A-module of the mannuronan C-5-epimerase AlgE4 from Azotobacter vinelandii. J Biol Chem.

[7] Whitfield G.B., Marmont L.S., Howell P.L. (2015). Enzymatic modifications of exopolysaccharides enhance bacterial persistence. Front Microbiol.

[8] Van Olst B., Eerden S.A., Eštok N.A., Roy S., Abbas B., Lin Y., van Loosdrecht M.C.M., Pabst M. (2025). Metaproteomic profiling of the secretome of a granule-forming ca. Accumulibacter enrichment. Proteomics.

[9] Dueholm M.K.D., Besteman M., Zeuner E.J., Riisgaard-Jensen M., Nielsen M.E., Vestergaard S.Z., Heidelbach S., Bekker N.S., Nielsen P.H. (2023). Genetic potential for exopolysaccharide synthesis in activated sludge bacteria uncovered by genome-resolved metagenomics. Water Res.

[10] Srivastava J., Sunthar P., Balaji P.V. (2020). The glycan alphabet is not universal: a hypothesis. Microb Genom.

[11] Schmid J., Sieber V., Rehm B. (2015).

[12] Kim N.K., Mao N., Lin R., Bhattacharyya D., van Loosdrecht M.C.M., Lin Y. (2020). Flame retardant property of flax fabrics coated by extracellular polymeric substances recovered from both activated sludge and aerobic granular sludge. Water Res.

[13] Gongi W., Pinchetti J.L.G., Cordeiro N., Sadok S., Ouada H. Ben (2022). Characterization of biodegradable films based on extracellular polymeric substances extracted from the thermophilic microalga graesiella sp. Algal Res.

[14] Saha I., Datta S., Biswas D. (2020).

[15] Nielsen P.H., McIlroy S.J., Albertsen M., Nierychlo M. (2019).

[16] Páez-Watson T., van Loosdrecht M.C.M., Wahl S.A. (2024). From metagenomes to metabolism: systematically assessing the metabolic flux feasibilities for “Candidatus Accumulibacter” species during anaerobic substrate uptake. Water Res.

[17] Chen L.M., Hofstra T., Langedijk J., Andrei S., Pabst M., Pronk M., van Loosdrecht M.C.M., Lin Y. (2025). Identification of a surface protein in the extracellular polymeric substances of seawater-adapted aerobic granular sludge. Water Res.

[18] Tomás-Martínez S., Zwolsman E.J., Merlier F., Pabst M., Lin Y., van Loosdrecht M.C.M., Weissbrodt D.G. (2023). Turnover of the extracellular polymeric matrix of granules performing biological phosphate removal. Appl Microbiol Biotechnol.

[19] Parks D.H., Imelfort M., Skennerton C.T., Hugenholtz P., Tyson G.W. (2015). CheckM: assessing the quality of microbial genomes recovered from isolates, single cells, and metagenomes. Genome Res.

[20] Petriglieri F., Singleton C.M., Kondrotaite Z., Dueholm M.K.D., McDaniel E.A., McMahon K.D., Nielsen P.H. (2022). Reevaluation of the phylogenetic diversity and global distribution of the genus “ Candidatus Accumulibacter”. mSystems.

[21] Katoh K., Standley D.M. (2013). MAFFT multiple sequence alignment software version 7: improvements in performance and usability. Mol Biol Evol.

[22] Price M.N., Dehal P.S., Arkin A.P. (2010). FastTree 2 - approximately maximum-likelihood trees for large alignments. PLoS One.

[23] Eddy S.R. (2011). Accelerated profile HMM searches. PLoS Comput Biol.

[24] Hyatt D., Chen G.-L., Locascio P.F., Land M.L., Larimer F.W., Hauser L.J. (2010). Prodigal: prokaryotic gene recognition and translation initiation site identification. http://www.biomedcentral.com/1471-2105/11/119.

[25] Aramaki T., Blanc-Mathieu R., Endo H., Ohkubo K., Kanehisa M., Goto S., Ogata H. (2020). KofamKOALA: KEGG ortholog assignment based on profile HMM and adaptive score threshold. Bioinformatics.

[26] Zhaxybayeva O., Gogarten J.P., Charlebois R.L., Doolittle W.F., Papke R.T. (2006). Phylogenetic analyses of cyanobacterial genomes: quantification of horizontal gene transfer events. Genome Res.

[27] Fitch W.M. (1971). Toward defining the course of evolution: minimum change for a specific tree topology. Syst Biol.

[28] Páez-Watson T., Tomás-Martínez S., de Wit R., Keisham S., Tateno H., van Loosdrecht M.C.M., Lin Y. (2024). Sweet secrets: exploring novel glycans and glycoconjugates in the extracellular polymeric substances of “Candidatus Accumulibacter.”. ACS ES&T Water.

[29] Rehm B.H.A. (2010). Bacterial polymers: biosynthesis, modifications and applications. Nat Rev Microbiol.

[30] Vorhölter F.J., Schneiker S., Goesmann A., Krause L., Bekel T., Kaiser O., Linke B., Patschkowski T., Rückert C., Schmid J., Sidhu V.K., Sieber V., Tauch A., Watt S.A., Weisshaar B., Becker A., Niehaus K., Pühler A. (2008). The genome of Xanthomonas campestris pv. campestris B100 and its use for the reconstruction of metabolic pathways involved in xanthan biosynthesis. J Biotechnol.

[31] Wang Q., Perepelov A.V., Feng L., Knirel Y.A., Li Y., Wang L. (2009). Genetic and structural analyses of Escherichia coli O107 and O117 O-antigens: research article. FEMS Immunol Med Microbiol.

[32] de Jong S., van de Velde F. (2007). Charge density of polysaccharide controls microstructure and large deformation properties of mixed gels. Food Hydrocoll.

[33] Arumugam K., Baǧcl C., Bessarab I., Beier S., Buchfink B., Górska A., Qiu G., Huson D.H., Williams R.B.H. (2019). Annotated bacterial chromosomes from frame-shift-corrected long-read metagenomic data. Microbiome.

[34] Oehmen A., Zeng R.J., Yuan Z., Keller J. (2005). Anaerobic metabolism of propionate by polyphosphate-accumulating organisms in enhanced biological phosphorus removal systems. Biotechnol Bioeng.

[35] Welles L., Tian W.D., Saad S., Abbas B., Lopez-Vazquez C.M., Hooijmans C.M., van Loosdrecht M.C.M., Brdjanovic D. (2015). Accumulibacter clades type I and II performing kinetically different glycogen-accumulating organisms metabolisms for anaerobic substrate uptake. Water Res.

[36] Christie M.R., McNickle G.G. (2023).

[37] Betts A., Kaltz O., Hochberg M.E. (2014). Contrasted coevolutionary dynamics between a bacterial pathogen and its bacteriophages. Proc Natl Acad Sci USA.

[38] Mostowy R.J., Holt K.E. (2018).

[39] Arnold J.W., Spacht D., Koudelka G.B. (2016). Determinants that govern the recognition and uptake of Escherichia coli O157 : H7 by Acanthamoeba castellanii. Cell Microbiol.

[40] Siler E., Friesen M.L. (2017). Widespread negative frequency-dependent selection maintains Diversity in the legume-rhizobia Symbiosis: balancing nodulation May explain the paradox of rhizobium diversity.

[41] Wang Q., Liu J., Zhu H. (2018).

[42] Hester S.E., Park J., Goodfield L.L., Feaga H.A., Preston A., Harvill E.T. (2013). Horizontally acquired divergent O-antigen contributes to escape from cross-immunity in the classical bordetellae. BMC Evol Biol.

[43] Iguchi A., Shirai H., Seto K., Ooka T., Ogura Y., Hayashi T., Osawa K., Osawa R. (2011). Wide distribution of O157-antigen biosynthesis gene clusters in Escherichia coli. PLoS One.

[44] Patil P.B., Bogdanove A.J., Sonti R.V. (2007). The role of horizontal transfer in the evolution of a highly variable lipopolysaccharide biosynthesis locus in xanthomonads that infect rice, citrus and crucifers. BMC Evol Biol.

[45] Van Kranenburg R., Marugg J.D., Van Swam I.I., Willem N.J., De Vos W.M. (1997). Molecular characterization of the plasmid-encoded eps gene cluster essential for exopolysaccharide biosynthesis in Lactococcus lactis. Mol Microbiol.

